# Characterization of Material Extrusion-Printed Amorphous Poly(Ether Ketone Ketone) (PEKK) Parts

**DOI:** 10.3390/polym17081069

**Published:** 2025-04-16

**Authors:** Thomas Hanemann, Alexander Klein, Siegfried Baumgärtner, Judith Jung, David Wilhelm, Steffen Antusch

**Affiliations:** 1 Institute for Applied Materials, Karlsruhe Institute of Technology, Hermann-von-Helmholtz-Platz 1, D-76344 Eggenstein-Leopoldshafen, Germany; a.klein@kit.edu (A.K.); siegfried.baumgaertner@kit.edu (S.B.); judith.jung@kit.edu (J.J.); david.wilhelm@gmx.net (D.W.); steffen.antusch@kit.edu (S.A.); 2 Department of Microsystems Engineering, University Freiburg, Georges-Koehler-Allee 102, D-79110 Freiburg, Germany

**Keywords:** material extrusion, MEX, fused filament fabrication, FFF, poly(ether ketone ketone), PEKK

## Abstract

Poly(ether ketone ketone) (PEKK), as a representative of high-performance poly(aryl ether ketones), shows outstanding thermomechanical properties, opening up a huge range of different applications in various technical fields. Its appearance as a quasi-amorphous polymer with a certain suppression of the crystallization process facilitates melt processing via additive manufacturing processes like material extrusion (MEX), especially in fused filament fabrication (FFF). The quality of the printing process is proven in this work by tensile testing and surface roughness measurements of suitable specimens. The MEX printing of semicrystalline PEKK faces two major challenges: on the one hand, the very high printing temperature is in contrast to established engineering plastics, and on the other hand, it is difficult to avoid crystallization after printing. The first issue can be addressed by using suitably enhanced MEX printers and the second one by selecting adapted printing parameters. The measured Young’s modulus (3.49 GPa) and tensile strength (104 MPa) values are higher than the related vendors’ data given for filaments (3.0 GPa and 92 MPa, respectively). In addition, the temperature-dependent thermal conductivity is determined, and the values of well-established PEEK (poly(ether ether ketone)) in the temperature range from 20 to 180 °C are mostly slightly higher in comparison to the related PEKK data. Based on the results, PEKK can be a useful substitute for well-established PEEK because of their comparable properties. However, PEKK has a pronouncedly lower FFF printing temperature, combined with a reduced tendency of the device to warp after printing. A larger printed test part with some surface structures shows the improved printability of PEKK in comparison to PEEK.

## 1. Introduction

High-performance polymers are gaining importance and extend the application range of plastics up to 250 °C and in some cases to even higher temperatures. The most important chemical structures are poly(phenyl ether) (PPE), poly(phenyle oxide) (PPO), poly(aryl sulfonones) (PSU), poly(ether imide) (PEI), poly(phenyle sulfide) (PPS), liquid crystalline polymer (LCP), and the huge group of aromatic poly(aryl ether ketones) (PAEK), with the most prominent representative being poly(ether ether ketone) (PEEK) [1]. The family of PAEK polymers has several advantages regarding their chemical and physical properties. This includes outstanding thermomechanical properties, creep resistance, and dimensional stability, even at elevated temperatures. Their continuous operation temperature is significantly higher, up to almost 300 °C, in contrast to widely used engineering polymers like PMMA, PC, PA, PVC, and others.

Their high thermomechanical stabilities, even at elevated temperatures, open up various application fields, where the different metals that have been used up to now may be substituted by high-performance polymers, like materials in the PAEK family [2]. As an example, rapid metal molding tools, e.g., those that are used in casting or injection molding and related technologies, can be made from polymers with a high continuous operating temperature [3]. Another possible field of application can be lightweight construction, e.g., for aviation or trains in non-critical areas, like luggage storage. Beyond thermomechanical issues, PAEKs, as high-performance polymers, show a high chemical resistivity against most organic solvents, as well as water and physiologic salt solutions. This enables their usage as medical implant materials [4,5,6,7,8,9,10]. Other modern fields of application are chemical and process equipment [8], tissue engineering or pharmaceutical engineering [9], and healthcare [10]. As a drawback, the advanced thermomechanical stability of the PAEK family is accompanied by more complicated melt processing for the fabrication of parts due to their very high softening and melting temperatures, which can only be addressed by using elaborated equipment for shaping. Beyond injection molding as an established polymer melt processing method for thermoplastics, additive manufacturing, especially fused filament fabrication (FFF) as part of the material extrusion family (MEX), is gaining importance. It can be used for the realization of customized devices, and it is a sustainable shaping method, owing to the significantly reduced material consumption. The FFF of high-performance polymers requires more sophisticated and expensive 3D printer equipment, like liquid-cooled printheads, due to the high printing process temperature of up to 500 °C, as in the case of PEEK [11] with its very high melting temperature of around 350 °C [6]. As a result, a very high continuous operation temperature, up to 260 °C, is possible, which allows for, e.g., their use as mold inserts carrying simple test structures for powder injection molding with highly filled alumina feedstocks [12]. Most high-performance polymers contain rigid aromatic moieties in the polymer chain, like aromatic ethers or ketones. This results in a semicrystalline phase behavior [7], which may cause pronounced warpage during cooling down after melt processing. This can be compensated for to some extent by suitable printing parameters and printed part designs [12]. Whilst many publications deal with the FFF printing of PEEK, only a few research groups investigate the FFF printing of PEKK [13,14,15,16,17,18,19,20], despite their comparable thermomechanical properties and the pronounced suppression of crystallization during cooling down from the melting process [13] (Table 1).

In an early work, Quiroga Cortes et al. investigated three different PEKKs of the Kepstan family (Arkema, Seoul, Republic of Korea) with different monomer ratios (terephthalyl (t)/isophthalyl (i) monomers) [17]. Increasing isophthalyl amounts lead to a suppression of the crystallization process and lower glass transition and melting temperatures [17]. Consequently, the melt processing is simplified. Paszkiewiecz et al. compared the usage of different PEEK and PEKK types, printed by FFF, as dental implants [13]. They applied the same Kimya PEKK-A filament as in the present study with a (t)/(i) ratio of 60/40. For comparison with the different PEEK samples, the PEKK specimens were treated thermally up to a temperature of 400 °C. In comparison to PEEK, better mechanical properties could be obtained, and less bacterial adhesion was observed. Maloney and coworkers investigated the impact of FFF printing parameters on flexural properties [16]. The used PEKK type has a different monomer composition (with a (t)/(i) ratio of 70/30). As a result, the crystallization rate exceeds that of the PEKK-A type that has a higher crystalline portion. Maloney et al. found a pronounced influence of the printing parameters, e.g., the printing speed, layer thickness, building platform, and nozzle temperature, on the degree of crystallinity in the printed specimen. Doyle et al. investigated slow-crystallizing PEKK-A and fast-crystallizing PEEK AM 200 [15]. They correlated the mechanical properties with the FFF printing parameters and thermal annealing. PEKK-A’s retention of the amorphous state during printing is advantageous in terms of mechanical properties. It can be attributed to better interlayer adhesion and a reduced number of pores [15]. A detailed investigation of the interlayer quality of FFF-printed PEKK-A samples was performed by Lepoivre and coworkers. They measured the viscosity and surface tension, used an IR camera for temperature recording during printing, and additionally carried out heat transfer modeling [18].

Currently, PAEK polymers are being discussed intensely for a wider application in different fields of medical technology, such as dentistry or facial surgery [19]. Rodzen and coworkers studied the influence of a hydroxyapatite filler in PEKK on the mechanical properties, crystallization behavior, as well as cell attachment of FFF-printed specimens [20]. Hong et al. compared titanium with titanium dioxide-filled PEKK implants, focusing on the osseointegration of facial implants [21]. Hydroxyapatite-coated FFF-printed PEKK can be used as a scaffold for tissue engineering in orthopedics [22]. FFF-printed mesh-type membranes made from PEEK and PEKK can be used for bone repair [23]. DFT simulations of its long-term performance can support the future application of PEKK as a dental implant material [24].

The crystallization of semicrystalline polymers like PEEK causes a pronounced distortion or warpage after melt processing by injection molding or FFF printing. Hence, the use of amorphous thermoplastics is recommended, especially for parts or potential products. The following sections will present the investigation of a commercial PEKK-A filament of a quasi-amorphous appearance with respect to its MEX printability, thermomechanical properties, and surface appearance after printing. The studies are aimed at facilitating printability compared to PEEK and at examining the potential use of PEKK-A as a material for an injection molding tool in a rapid tooling approach.

## 2. Materials and Methods

### 2.1. Material Selection

Compared to other available PEKK types and PEEK, the selected commercial pseudo-amorphous PEKK-A type, based on the Arkema Kepstan R 6000 (Arkema France, La Defense, France) [25,26] from Kimya, has the advantage of a very slow or even suppressed crystallization when the material is subjected to melt processing under standard conditions [15,16]. According to the vendors’ data, this was achieved by tailoring the ratio of the two used monomers containing terephthaloyl (60%) and isoterephthaloyl (40%) moieties within the copolymer’s composition. The most relevant PEKK properties are described in Table 1, and the data were taken from the vendors’ datasheets or from the webpage [25,26].

### 2.2. MEX Printing Parameter Selection

According to the vendors’ recommendation and after first test trials, all relevant printing parameters for both tensile properties and thermal conductivity are listed in Table 2. The selected printing parameters are in the range defined by Kimya. All MEX printing trials were performed using the APIUM P220 printer (Karlsruhe, Germany) with a printhead nozzle of 0.4 mm in diameter, an infill of 100%, and a layer thickness of 0.1 mm. As the description of the Apium P220 printer is no longer accessible on the webpage of the vendor [27], the most relevant characteristics are listed in Table 3. In contrast to other MEX printers for common thermoplastics like ABS or PLA, the x,y-resolution is relatively poor. Quite exceptional is the maximum printing temperature of 540 °C of the water-cooled printhead, which enables the MEX printing of high-performance thermoplastics in a wide temperature range. The selected printing temperature of 375 °C is higher than that of 340 °C reported in [13]. The printing speed is identical, as is the nozzle diameter. In the case of PEEK, the nozzle temperature was varied between 420 and 485 °C [11]. In [14], a larger nozzle (0.5 mm) was selected. The selected printing speed differs for the envelope (0.25 mm/s) and infill (30 mm/s). The printing temperature is almost identical at 370 °C.

### 2.3. Thermal Treatment

The MEX printing of semicrystalline thermoplastics like PEKK or PEEK benefits from a temperature-controlled chamber [12]. Unfortunately, the used APIUM P220 does not possess such a chamber. To induce crystallization, some printed samples were subjected to annealing in an oven (Mihm-Vogt GLP6, Stutensee, Germany). An initial heating up to 150 °C (heating rate 1 °C/min), with a dwell time of 30 min at 150 °C, was followed by a second temperature increase up to 200 °C, with a dwell time of 60 min at the maximum. To initiate crystallization, the temperature was lowered slowly to 150 °C within 60 min and then cooled to an ambient level, with a cooling rate of 1 °C per minute. This procedure was based on [13], with the final high-temperature treatment according to [17] being omitted. A similar temperature annealing program was used for the PEKK type in [15] with the same maximum temperature of 200 °C, followed by a slow gradient (1 °C/min) down to an ambient temperature.

### 2.4. Sample Characterization

**Figure 1:**
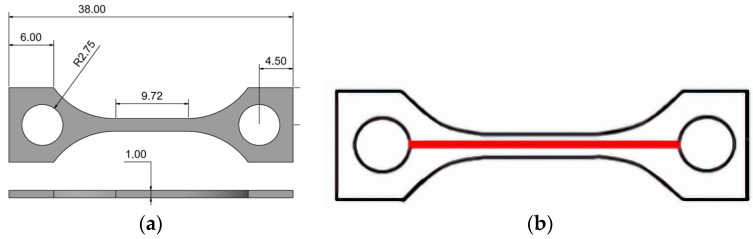
(**a**) Tensile test specimen geometry (mm); (**b**) measured length on the tensile test specimen for surface roughness identification.

Tensile testing was carried out in accordance with DIN EN ISO 527-1 [28] using a universal testing machine Z 100 (Zwick-Roell GmbH, Ulm, Germany) equipped with a 20 kN load cell and PMA 13/V7/1 (Maytec Mess- und Regeltechnik GmbH, Singen, Germany) extensometer. The specimen’s geometry was selected according to the standard described above (Figure 1a). The sample thickness was 1 mm, and the sample width was 11.5 mm. The testing speed was set to 50 mm/min. All measurements were performed at 20 °C. Fracture analysis was carried out via SEM (Zeiss Gemini, Zeiss Microscopy GmbH, Oberkochen, Germany). To increase the conductivity, the fracture area was sputtered with gold (Emitech K575, Quorum Technologies Ltd., Laughton, UK). The surface roughness was measured by a white light interferometer (MicroProf^®^ CWL F, FRT GmbH, Bergisch-Gladbach, Germany) according to the DIN EN ISO 4287 standard [29]. Five samples of each were investigated. The measured length was 22 mm, with an optical resolution of 1 µm and a sample rate of 32 Hz (Figure 1b). Thermal conductivity was measured by DSC (dynamic scanning calorimetry, heating/cooling rate 10 K/min, −10–200 °C, argon atmosphere, three runs, Netzsch DSC 204 (Netzsch Gerätebau, Selb, Germany), (sapphire as reference, experimental error ±2.5%), combined with a laser-flash method (range 25–180 °C, 2 measurements, Netzsch LFA 427 (Netzsch Gerätebau, Selb, Germany), experimental error ±3.0%). The disk-shaped samples for the laser-flash method had a diameter of 12.7 mm and a height of 0.8 mm. The densities of the samples were measured in 2-propanol according to Archimedes’ method. A precision balance (Secura 225D-1S equipped with YDK 01, Sartorius Lab Instruments GmbH & Co., KG, Göttingen, Germany, experimental error ±2.5%) was applied.

## 3. Results and Discussion

### 3.1. MEX Printing of Tensile Test Specimens

**Figure 2:**
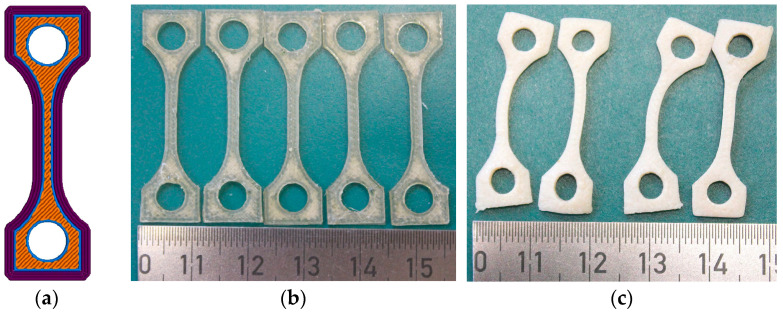
(**a**) Schematic drawing of the infill orientation (45°) and number and size of the contour lines of envelopes [8]; (**b**) printed tensile test specimens prior to thermal treatment; (**c**) printed tensile test specimens after thermal treatment.

Based on previous investigations of PEEK and PEEK-based composites [11], an infill orientation tilted by 45° relative to the long axis of the tensile test specimen was selected to prevent anisotropic properties. The outer contour was generated by three envelope filament depositions (Figure 2a). The printed specimens prior to thermal treatment are shown in Figure 2b. The traces caused by the printhead movement are clearly visible, as is the good optical transmittance, which reflects retention of the amorphous polymer state. The thermal treatment after printing as described in Section 2.3 caused pronounced specimen distortion and warpage (Figure 2c). Hence, tensile testing was impossible. Due to the surface’s whitish appearance, a semicrystalline polymer state resulting from annealing [15] can be deduced. In contrast to a glass transition, the crystallization process is accompanied by an instantaneous volume reduction, which causes inner stress that induced sample warpage, as well as void formation.

### 3.2. Printed Sample Characterization

#### 3.2.1. Mechanical Testing

**Figure 3:**
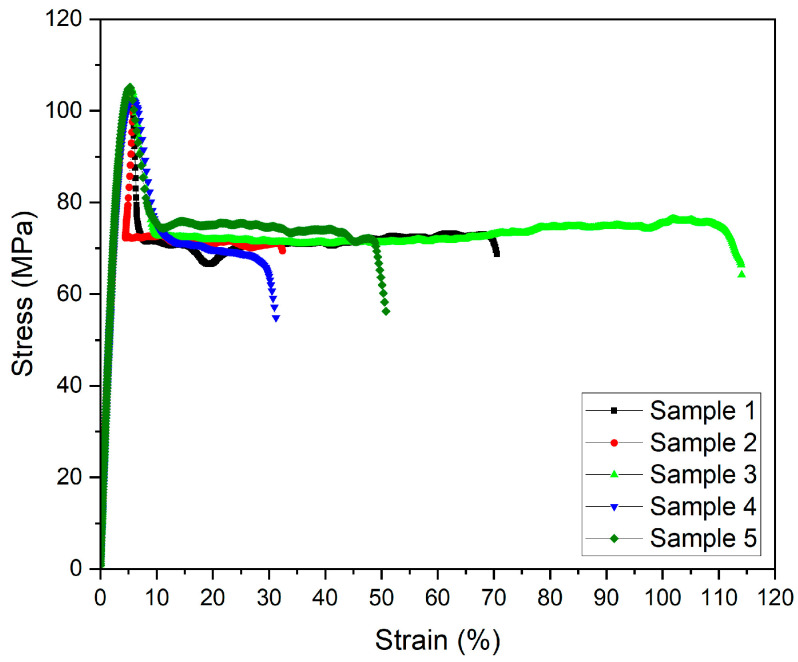
Stress–strain diagrams of printed PEKK samples.

The mechanical testing results, here the stress–strain correlation, measured at 20 °C for five printed samples with identical printing parameters are visualized in Figure 3. These samples were not subjected to thermal treatment. Unfortunately, the elongation behavior scatters broadly, which may be attributed to a non-unique degree of crystallinity of the non-annealed samples. Despite the wide scattering of the elongation at break values, the other relevant parameters exhibit an acceptable standard deviation. An almost similar non-systematic behavior was observed by Paszkiewicz et al., with elongation at break values between 5 and 120% representing the range from brittle to ductile behavior [13].

Doyle et al. found elongation at break values of below 20% and more than 100% for amorphous PEKK [15]. They attributed the high elongation at break values to the presence of small pores between the printed layers, which caused layer-to-layer slipping and, as a consequence, higher strains [15]. The measured Young’s modulus and tensile strength (Table 4) are higher (by 17% and 13%, respectively) than the filament values provided by the vendor (Table 1) and reported in reference [13]. These values can be interpreted as an indication of the absence of large voids in the printed test specimen.

Doyle et al. [15] reported tensile strength values of 84 MPa (printing direction x-axis) and 69 MPa (printing direction z-axis) and Young’s moduli of 3.2 GPa (x-axis) and 2.5 GPa (z-axis), which are lower than the data presented here. They used a similar printing speed (envelope 25 mm/s; infill 30 mm/s) and a layer thickness of 0.25 mm, which is significantly higher than the 0.1 mm applied in this case. A printing temperature of 370 °C was selected [15]. It has been reported that smaller layer thicknesses cause enhanced mechanical properties [15]. Maloney et al. stated that the flexural modulus increased by 17% when the printed layer thickness was reduced from 0.2 mm to 0.1 mm [16]. Doyle et al. reported a small void fraction in the x,y-direction of 1.5% but a high void fraction in the z-direction of 6%. The total volume of voids in the sample directly affects the resulting bulk mechanical properties [15]. The tilting of the isotropic printing direction by 45° in combination with the above printing parameters, including the higher printing temperature (375 °C) and the smaller layer thickness, might have reduced the typical FFF voids in the sample. This might have enhanced the mechanical stability, as reflected by the higher Young’s modulus and tensile strength values.

**Figure 4:**
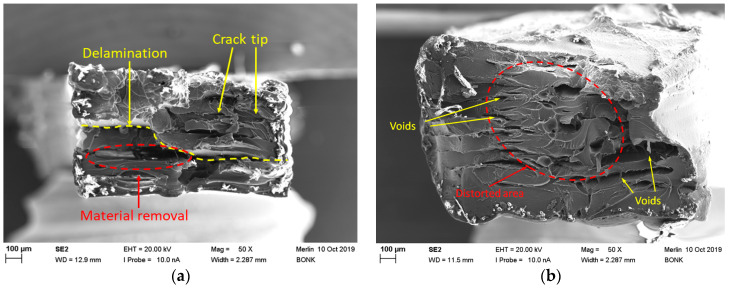
Fracture images of (printing layer thickness 0.1 mm) (**a**) printed tensile test specimen without thermal treatment and (**b**) thermally treated printed sample, broken manually.

To investigate the influence of annealing on the semicrystalline appearance, a printed tensile testing sample was broken manually. The resulting fracture image was compared with that of a non-annealed quasi-amorphous sample after tensile testing (Figure 4). In both samples, individual layers originating from the printing process can be detected. The amorphous sample (Figure 4a) exhibits a small number of tiny voids and a torn volume element (lower left part). In the center of the temperature-treated sample, a pronounced distorted area indicates the presence of a semicrystalline domain due to pronounced shrinkage during crystallization (Figure 4b). In contrast to this, the fracture images of pure PEKK reported in reference [14] show a pronounced presence of typical FFF-diamond-shaped voids, despite the reported infill of 100%. The CT images in [15] show a high number of voids in the printed samples, which may explain the lower Young’s modulus and tensile strength. Quite recently, Maloney et al. presented fracture images of samples with the same layer thickness of 0.1 mm, which showed a remarkable number of voids [16].

#### 3.2.2. Surface Roughness

The different surface roughness values were measured in five printed test specimens, with the test lengths being shown in Figure 1b (Table 5). The listed roughness values are defined as follows:

- R_max_: The maximum difference between the valley and peak.
- R_z_: The maximum peak-to-valley height of the measured profile line.
- R_a_: The average deviation from the mean or center line.
- R_q_: The average root mean square deviation from the mean line.

Compared to previously published results for pure PEEK with the same test structure (Figure 1b), the surface roughness values are significantly smaller, which is favorable for, e.g., the use as mold inserts in polymer melt processing [11,12].

#### 3.2.3. Thermal Conductivity

The estimation of thermal conductivity requires measurements of three different parameters: the density, temperature conductivity, and specific heat capacity. For simplification and due to restrictions resulting from Archimedes’ method, the density value was only measured at 25 °C (1.24 g/cm^3^). The thermal conductivity *λ* can be calculated according to Equation (1) (*ρ*: density at 25 °C; *C_p_*: specific heat capacity; *α*: temperature conductivity (thermal diffusivity)).

(1) $$\lambda =\alpha \;\rho \;C_{p\;}$$

**Figure 5:**
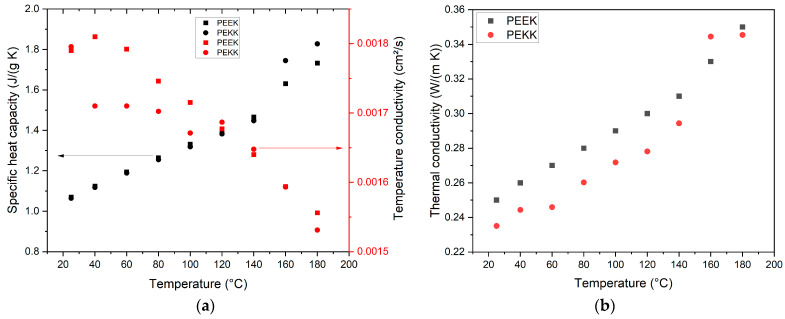
Thermal conductivity measurements: (**a**) specific heat capacity and temperature conductivity as function of temperature measured by DSC and laser flash method; (**b**) temperature-dependent thermal conductivity. PEEK data are taken from [11].

Figure 5 shows the measured change in the specific heat capacity and temperature conductivity (Figure 5a), as well as the resulting thermal conductivity of PEKK and, for comparison, of PEEK (Figure 5b). The latter values were partly taken from [11]. Up to 140 °C, the PEEK values are slightly higher than the corresponding PEKK values, which can be attributed to the semicrystalline appearance of PEEK in contrast to the quasi-amorphous PEKK. Only at 160 °C is this order changed. According to Equation (1), the thermal conductivity is calculated from the material’s density, heat capacity, and thermal diffusivity. In the temperature range between 140 and 165 °C, both materials exhibit a phase transition (PEEK: 143 °C; PEKK: 159 °C). The measuring point at 160 °C is very close to the phase transition of PEKK, as given in the datasheet (159 °C) or measured more accurately by Quiroga Cortes et al. (155–158 °C, depending on the cooling rate) [17]. At a phase transition, the heat capacity value is not constant anymore. This may explain the higher thermal conductivity of PEKK compared to PEEK. Far away from the phase transition at 180 °C, PEEK’s thermal conductivity is higher.

### 3.3. MEX Printing as a Rapid Tooling Method

**Figure 6:**
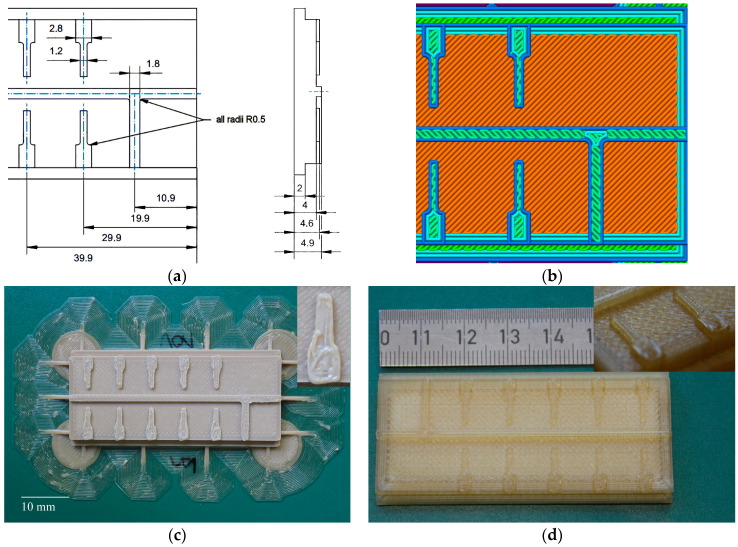
Mold inserts with fluidic test structures (mm): (**a**) a schematic drawing; (**b**) excerpt of the used STL file; (**c**) a printed PEEK part with a structural detail image inserted; (**d**) a printed PEKK part with a structural detail image inserted.

Rapid tooling uses rapid prototyping methods like FFF and enables cost-efficient testing of mold inserts. In addition, it allows for small-scale fabrication by polymer melt processing, with examples including different injection molding processes and powder injection by applying highly filled feedstock with, e.g., 50 vol% alumina as a ceramic filler. Previously published work [3,11,12] focused on PEEK and PEEK-derived composites and their potential use as mold insert materials in polymer replication. Considering the requirements that must be met by a mold insert in polymer melt processing, such as enhanced mechanical stability, maximum continuous operation temperature, low surface roughness, and good thermal conductivity, the polymers of the PAEK family are very promising [12]. PEKK-A is a suitable mold insert material, as it combines good material properties with simplified FFF printing due to crystallization and device warpage being prevented. The relevant printing parameters for PEKK and, for comparison, PEEK are listed in Table 6. Compared to the tensile test specimen, the extruder temperatures are increased for enhanced structure filling and viscosity reduction. The printing temperature of PEEK is still significantly higher than that of PEKK. The printing speed is reduced to reach an improved printing quality of structural details. When producing a ceramic microreactor, the mold insert has a far more complex structure, as it accommodates the negative fluid structures (long channels) and the structures for electrode positioning (free-standing wall with a shaft) (Figure 6a) [3]. The latter have a height of 0.6 mm and a width of 1.2 mm. The width of the inner channels is 1.8 mm, and the total width of the mold insert is 30 mm.

Figure 6b shows an excerpt of the generated STL file covering the envelope and infill trails. The shaft of the fine structures consists of two envelope trails, the tip is made by one trail only, and the remaining inner area is completed by the infill. The printing of these structural details suffers from the limited Apium P220 resolution (Table 3) and the pronounced shrinkage and device warpage after crystallization in the case of PEEK (Figure 6c). The integration of auxiliary structures like a large brim was mandatory to fix the printed part that was to the built plate during printing to prevent delamination. Especially the insert in Figure 6c reveals the poor surface and outer contour quality. The related structure in PEKK (Figure 6d) showed an improved quality of both the surface and outer contour. Auxiliary structures like brims were not needed due to the significantly reduced part warpage. Despite the presented significant improvements, the surface quality is still poor compared to a structure generated by the PolyJet^R^ technology with its superior performance, as presented in an earlier work [3]. Unfortunately, the thermosets that were generated in the PolyJet^R^ printing process possess softening temperatures between 50 and 60 °C, which are critical in replication at high temperatures and powder injection molding in the long run. The general FFF printability and the surface appearance and quality of a printed part are mainly influenced by the printing parameters, the melt viscosity, wetting on the previously printed polymer layer, the surface tension, and thermal properties. A clear correlation with the chemical structure, which indirectly affects the listed material properties, can hardly be made.

## 4. Conclusions and Future Outlook

This report shows that PEKK can be printed via FFF with an acceptable quality and that it has the potential to be a suitable alternative to PEEK in certain applications. The quasi-amorphous PEKK-A presented here allows for better processing during and after printing, as crystallization is prevented, resulting in non-warped printed samples. Especially the significantly lower printing temperature compared to PEEK facilitates the MEX printing process. The tensile testing results demonstrate scatter over a large range. This was observed elsewhere as well. The measured mechanical properties, such as Young’s modulus and tensile strength, were higher than the data reported in the literature, which can be attributed to a different MEX printing strategy causing a lower number of internal defects like voids. With respect to their application as plastic mold inserts in rapid tooling, the printed PEKK samples showed a smoother surface and allowed for improved structural detailing. This is also a consequence of the better material behavior during printing. The surface quality, however, was still lower than that of tools fabricated by the PolyJet technology. The thermal conductivity measured over a wide temperature range is slightly lower than the values for PEEK, but in agreement with data taken from literature.

Future work should focus on enhancing the reproducibility by, e.g., a systematic thermal treatment and further optimization of the printing parameters to improve the surface quality.

## Figures and Tables

**Table 1:** A comparison of the thermomechanical properties of PEEK and PEKK, taken from the vendors’ datasheets.

ITEM	PEEK ^1^	PEKK ^2^
Density (g/cm^3^)	1.32	1.26
Moisture absorption (%)	0.03	0.29
Flexural modulus (MPa)	3.6 (xy); 3.7 (xz)	2.6 (xy); 2.2 (xz)
Elongation at break (%)	19.1 (xy); 16.1 (xz)	5.6 (xy); 4.0 (xz)
Tensile strength (MPa)	98.3 (xy); 93.7 (xz)	92.1 (xy); 76.9 (xz)
Young’s modulus (GPa)	4.0 (xy); 3.7 (xz)	3.0 (xy); 2.7 (xz)
Glass transition temperature (°C)	143	159
Melting temperature (°C)	343	308
Heat deflection temperature (°C)	162 (HDT-A)	139 (HDT-A)
Thermal conductivity (W/(m K))	0.25	0.21

^1^ Vendors’ datasheet: https://www.ensingerplastics.com/en/filaments/tecafil-peek-vx-natural-1-75-mm#/product-technical-detail-collapse-item-2-lvl-1, accessed on 20 March 2025. ^2^ Vendors’ datasheet: https://get3d.pl/wp-content/uploads/2020/09/kimya_fiche_PEKK-A_en_GB.pdf, accessed on 20 March 2025.

**Table 2:** MEX printing parameters for tensile testing and thermal conductivity measurement.

ITEM	PEKK (Vendors’ Recommendation ^1^)	PEKK (Own Parameters)
Extruder temperature (°C)	370–380	375
Printing speed (mm/s)	20–40	30
Infill orientation (°)	45	45

^1^ Vendors’ recommendation: https://get3d.pl/wp-content/uploads/2020/09/kimya_fiche_PEKK-A_en_GB.pdf; accessed on 20 March 2025.

**Table 3:** Most relevant characteristics of the APIUM P220 MEX printer.

ITEM	Specification
x,y-resolution (mm)	0.5
z-resolution (mm)	0.1
Smallest layer height (mm)	0.1
Smallest wall thickness (mm) (@0.4 mm nozzle)	0.5
Reproducibility (mm)	0.1
Standard nozzle diameter (mm)	0.4
Filament diameter (mm)	1.75
Max. temperature of built platform (°C)	160
Max. printhead temperature (°C)	540

**Table 4:** Average data on all measured mechanical properties.

Material	Young’s Modulus (GPa)	R_p0.2_ (MPa)	Tensile Strength Rm (MPa)	Elongation at Max. Force (%)	Ultimate Tensile Strength (MPa)	Elongation at Break (%)
PEKK	3.5 ± 0.2	59.4 ± 5.4	104 ± 1.2	5.4 ± 0.2	63 ± 6.9	61 ± 36

**Table 5:** Summary of all surface roughness values measured for amorphous PEKK and PEEK, printed at 420 °C [11].

Material	R_max_ (µm)	R_z_ (µm)	R_a_ (µm)	R_q_ (µm)
PEKK	79.7 ± 17.6	62.7 ± 19.2	9.0 ± 0.6	11.7 ± 1.1
PEEK [8]	141	122	16	n.a.

**Table 6:** Relevant MEX printing parameters for mold insert printing.

ITEM	PEEK	PEKK
Nozzle diameter (mm)	0.4	0.4
Extruder temperature (°C)	430	360
Printing speed (mm/s)	10	10
Infill orientation (°)	45	45

## Data Availability

The raw data supporting the conclusions of this article will be made available by the authors on request.
